# Abnormal development of sensory-motor, visual temporal and parahippocampal cortex in children with learning disabilities and borderline intellectual functioning

**DOI:** 10.3389/fnhum.2014.00806

**Published:** 2014-10-15

**Authors:** Francesca Baglio, Monia Cabinio, Cristian Ricci, Gisella Baglio, Susanna Lipari, Ludovica Griffanti, Maria G. Preti, Raffaello Nemni, Mario Clerici, Michela Zanette, Valeria Blasi

**Affiliations:** ^1^IRCCS, Don Carlo Gnocchi Foundation – ONLUSMilan, Italy; ^2^Department of Pathophysiology and Transplantation, University of MilanMilan, Italy; ^3^Department of Epidemiology and Preventive Medicine, University of RegensburgGermany; ^4^Department of Electronics, Information and BioengineeringPolitecnico di Milano, Milan, Italy

**Keywords:** borderline intellectual functioning (BIF), motor development, learning disabilities, brain maturation, voxel-based morphometry (VBM)

## Abstract

Borderline intellectual functioning (BIF) is a condition characterized by an intelligence quotient (IQ) between 70 and 85. BIF children present with cognitive, motor, social, and adaptive limitations that result in learning disabilities and are more likely to develop psychiatric disorders later in life. The aim of this study was to investigate brain morphometry and its relation to IQ level in BIF children. Thirteen children with BIF and 14 age- and sex-matched typically developing (TD) children were enrolled. All children underwent a full IQ assessment (WISC-III scale) and a magnetic resonance (MR) examination including conventional sequences to assess brain structural abnormalities and high resolution 3D images for voxel-based morphometry analysis. To investigate to what extent the group influenced gray matter (GM) volumes, both univariate and multivariate generalized linear model analysis of variance were used, and the varimax factor analysis was used to explore variable correlations and clusters among subjects. Results showed that BIF children, compared to controls have increased regional GM volume in bilateral sensorimotor and right posterior temporal cortices and decreased GM volume in the right parahippocampal gyrus. GM volumes were highly correlated with IQ indices. The present work is a case study of a group of BIF children showing that BIF is associated with abnormal cortical development in brain areas that have a pivotal role in motor, learning, and behavioral processes. Our findings, although allowing for little generalization to the general population, contribute to the very limited knowledge in this field. Future longitudinal MR studies will be useful in verifying whether cortical features can be modified over time even in association with rehabilitative intervention.

## INTRODUCTION

In the last decades great attention has been devoted to specific learning disabilities leading to a significant improvement in diagnosis, treatment, and also detection of the neuroanatomical bases of these conditions. Among the school age children population, learning difficulties are very frequent, but the majority of these children do not meet the diagnostic criteria for specific learning disorders and fall into the non-specific learning disability category. In this group of children the great majority meet criteria for borderline intellectual functioning (BIF), which is a complex clinical entity that has received very little scientific investigation.

Borderline intellectual functioning has been described by the Diagnostic and Statistical Manual (DSM-IV-TR; American Psychiatric Association, 2000) to describe an intelligence quotient (IQ) range that is between 1 and 2 SD below the mean (IQ: 70–85) and it is conceptualized as the frontier that delimits normal intellectual functioning from intellectual disability (American Association of Mental Retardation [AAMR], 2002). According to recently published guidelines, BIF is a meta-condition characterized by heterogeneous cognitive difficulties, with a borderline IQ (between 71 and 85), and a deficit in personal functioning affecting daily and social activity (Salvador-Carulla et al., 2013). Its prevalence among school-aged population is estimated to be around 7% (Karande et al., 2008). BIF is not a single neurodevelopmental syndrome and for this reason it is not possible to trace back to a single functioning profile (Salvador-Carulla et al., 2013). The heterogeneity in the clinical profile of BIF is also reflected in the many different factors contributing to this condition: genetic liability; biological causes, i.e., perinatal adverse events, and epigenetic factors such as socioeconomic status (SES) and maternal stress (Aicardi, 1998; Bradley and Corwyn, 2002; Marcus Jenkins et al., 2014). Nevertheless, the majority (about 60%) of BIF children have a normal clinical profile regarding perinatal history, milestones, physical appearance, physical health, and middle class SES (Karande et al., 2008). BIF is a lifelong condition with obvious drawbacks in terms of social and vocational opportunities and health outcomes (Karande et al., 2008; Blacher et al., 2009; Emerson et al., 2010). Moreover, strong associations between borderline IQ and mental disorders in adult age (e.g., antisocial personality disorder, depression, suicide, and substance abuse) have been demonstrated (Douma et al., 2007; Ali and Hassiotis, 2008; Ferrari, 2009; Koenen et al., 2009; Emerson et al., 2010, 2011; Hassiotis et al., 2011).

Borderline intellectual functioning children typically present with intellectual (e.g., learning, reasoning, and problem solving) and adaptive skill difficulties (e.g., age-inappropriate social, communication, and daily living behaviors; Gresham and Elliott, 1987) and often show some degree of motor skill limitations (Frey and Chow, 2006; Fernell and Ek, 2010; Vuijk et al., 2010). Although BIF children have learning difficulties, these are not confined to a specific domain such as reading and writing (Karande et al., 2008). Moreover, BIF children often show some degree of written and spoken language comprehension deficits (Salvador-Carulla et al., 2013). With specific learning disorders they share poor performances in working memory (WM) and in short term memory (STM) tasks and consequent slowness in the execution of tasks requiring these abilities. Moreover, unlike specific learning disorders the adaptive skills limitation in social and academic areas of BIF children and most of all their learning difficulties reflect general intellectual functioning limitations with problems in attention, executive functions, gross, and fine motor abilities with lack of compensatory strategies (Cornoldi et al., 2014).

The advent of magnetic resonance imaging (MRI) allowed the quantification and visualization of structural brain changes *in vivo*, using specific morphometric techniques such as voxel-based morphometry (VBM; Ashburner and Friston, 2000), and FreeSurfer (Fischl et al., 1999), making feasible the investigation of brain structure also during development. In particular, the VBM technique is a widely used automatic technique that enables whole-brain analyses without a-priori hypotheses permitting the identification of structural differences between brains. A growing number of MRI studies investigated both normal (Giedd et al., 1999; Wilke and Holland, 2003; Sowell et al., 2004; Shaw et al., 2006) and abnormal (Sporn et al., 2003; Ridler et al., 2006; Paus et al., 2008) brain changes during development, showing that the majority of neuropsychiatric disorders are associated with deviations from normal brain development during childhood and/or adolescence. Despite the high prevalence and great social impact of BIF, there are no studies that investigated the brain characteristics in children with BIF using MRI.

The aim of this study was to determine if differences between BIF and typically developing (TD) children in terms of IQ level are also reflected in differences in gray matter (GM) brain volume and to identify factors able to explore for multiple correlations among these variables (IQ levels and GM volumes). To achieve this goal we collected brain MI structural images and IQ scores in 13 BIF children and 14 TD children. To limit the heterogeneity of the sample, we focused on BIF children with learning disabilities without the presence of genetic syndromes and/or major neuro-psychiatric disease, such as autism spectrum disorder or attentional deficit hyperactivity disorder (ADHD).

Due to the small number of subjects included in this study our study represents a case study of a group of BIF children with learning disabilities, allowing for little generalization of our findings to the general population of children with this condition, but contributing, for the first time, to the very limited knowledge in this field.

## MATERIALS AND METHODS

### PARTICIPANTS

Twenty-three children with BIF referred to the Adolescence and Pediatric Neuropsychiatry Unit of our Institute in the year 2011 were enrolled. All BIF children were referred to our Unit from mainstream schools due to their learning difficulties.

Children were diagnosed as BIF according to the American Psychiatric Association’s (2000). IQ scores, determined by means of the Wechsler Intelligence Scale for Children-III (WISC-III; Wechsler, 1991, 2006) ranged from 70 to 85 (mean Full Scale IQ score: FSIQ 80.26 ± 4.15). All BIF children underwent a clinical evaluation in order to exclude a neuropsychiatric disorder (such as ADHD and autism spectrum disorder), neurological conditions (epilepsy and traumatic brain injury), malformations or systemic diseases. All children included in the study had never taken medications, particularly referring to current or past psychostimulants, antidepressants, benzodiazepines and/or, antiepileptic drugs. None of the children included in the study were affected by genetic syndromes such as Down syndrome or Fragile X syndrome, nor had a positive history for systemic diseases, such as diabetes or immune disorders, nor infectious disease involving the central nervous system. Moreover, a detailed anamnesis that included pregnancy and perinatal history, developmental milestones, and associated disabilities (motor and language development and adaptive behavior) was collected for each BIF individual. All BIF children underwent a full learning abilities examination: reading and writing abilities were tested through a battery for developmental dyslexia and dysorthographia evaluation (DDE-2; Sartori and Job, 2007) and a reading test for primary school (Cornoldi and Colpo, 1998); visuo-spatial and motor integration skills were assessed using the developmental test of visual motor integration (VMI; Beery and Buktenica, 2000), while mathematic reasoning and calculation were tested with a battery for developmental dyscalculia (BDE; Biancardi and Nicoletti, 2004) or an assessment of math calculation and problem solving (AC-MT 6-11; Cornoldi et al., 2005). BIF children also underwent a full neuropsychological evaluation by means of: Neuropsychological Evaluation Battery for children (BVN 5–11; Bisiacchi et al., 2005); the Test of Reception of Grammar (TROG; Bishop, 2003); Modified Barrage bell test (Biancardi and Stoppa, 1997); Tower of London (TOL; Shallice, 1982; Sannio Fancello et al., 2006). Emotional and behavioral problems were assessed using the Child Behavioral Checklist (Achenbach and Rescorla, 2001, 2007).

The control group included fourteen TD children (M:F/7:7), with no history of neurological, psychiatric, or systemic disease or learning disability, selected from mainstream schools to be group matched to the BIF individuals for sex and age.

All children underwent a full IQ assessment by means of the WISC-III scale within 2 weeks of the MRI session and assessment of the socioeconomic status (SES; by Hollingshead’s (2011) Index of Social Position). The age range of recruited subject was 7–14 years for both groups. **Table 1** summarizes demographic characteristics, as well as IQ scores and SES, while **Table 2** shows the clinical data for each BIF child. The present study was approved by the scientific and ethics committees of our institution. All parents gave written informed consent for participation in the study.

**Table 1:** Demographic characteristics of BIF and TD groups and neuropsychological evaluation of BIF.

	BIF	TD	Group comparison
**Demographic**
*N*	13	14	*p-value*
*Age* (in years) [median (IQR)]	9.00 [8.56–9.62]	9.50 [8.50–11.75]	0.090^#^
*Sex* [M/F]	8/5	7/7	0.963^§^
*SES* [median (95%CI)]	3 (1.53–3.47)	4 (3–4.10)	0.008^#^
*WISC-III score*			
FSIQ [median (IQR)]	80 [77–83]	118 [107–120]	<0.0001^#^
VIQ [median (IQR)]	84 [77–86]	113.5 [103.25–118.25]	<0.0001^#^
PIQ [median (IQR)]	82 [79–85]	116 [105–118]	<0.0001^#^
**Neuropsychological assessment**
**BIF: *N* = 13**			*N*(*)
*Short term memory*
BVN 5-11 forward digit span [median (IQR)]	–0,66 [–1,64–1,09]	2/13
BVN 5-11 backward digit span [median (IQR)]	–0,30 [–2,12–0,70]	3/13
BVN 5-11 spatial span [median (IQR)]	–0,75 [–2,55–1,50]	4/13
*Long term memory*
BVN 5-11 word immediate recall [median (IQR)]	–0,59 [–3,65–1,68]	5/13
BVN 5-11 word delayed recall [median (IQR)]	0,39 [–4,45–1,76]	2/13
BVN 5-11 verbal paired associates learning [median (IQR)]	–0,68 [–2,15–0,58]	6/13
*Language*
BVN 5-11 naming [median (IQR)]	–1,00 [–1,80–0,96]	7/13
TROG-2 [median (IQR)]	92,00 [64–111]	3/13
*Executive functions and attention*
BVN 5-11 phonemic fluency [median (IQR)]	–0,45 [–2,77–0,86]	3/13
BVN 5-11 category fluency [median (IQR)]	–0,89 [–1,69–0,61]	6/13
TCM rapidity [median (IQR)]	–1,10 [–2,40–0,40]	9/13
TCM accuracy [median (IQR)]	–0,56 [–2,82–1,89]	4/13
TOL full score [median (IQR)]	–1,00 [–3,72–2,03]	7/13
*Socio-emotional level*
CBCL 6-18-Full Scale score [median (IQR)]	58 [34–69]	6/13
CBCL 6-18 internalizing [median (IQR)]	59 [33–69]	6/13
CBCL 6-18 externalizing [median (IQR)]	50 [32–59]	0/13

IQR, Interquartile range; SES, socioeconomic status by Hollingshead Index of Social Position; WISC-III, Wechsler Intelligent Scale for Children-III (standard score); FSIQ, full scale IQ; VIQ, verbal IQ; PIQ, performance IQ; BVN 5–11, Neuropsychological Evaluation Battery for Children 5–11 years old (*z*-score); TROG-2, the Test of Reception Of Grammar-2 (standard score); TCM, modified barrage bells test (*z*-score); TOL, Tower of London (*z*-score); CBCL, Child Behavioral Check-List 6–18 (*T*-score). *N*(*), Number of subjects with borderline or pathological score. Data analyzed by chi-square (§) or Mann–Whitney *U*-test ( ^#^).

**Table 2:** Demographic, anatomical MR, and clinical characteristics of BIF children.

Subject, sex	Conventional MR (brain abnormalities)	Pregnancy and perinatal history	Developmental milestones [MD/LD]	Comorbidity [L/M/B]	SES
1, M	–	GE: 34	LD	L	3
2, M	CC: Body, splenium	–	MD	L/M/B	3
3, F	–	–	MD	L/M/B	2
4, M	WMHs	–	LD	L/B	2
5, M	VE	GE: 30 + 5 (1080 g at birth); CS (oligohydramnios); NIC (discharge at 2 m, 11 d)	MD/LD	L/M/B	4
6, F	–	CS (risk of pre-eclampsia); NIC (discharge at 5 d)	–	M	4
7, M	–	CS (umbilical cord around neck); APGAR 3/8	LD	L/M	4
8, M	–	GE: 37; CS (abruptio placentae)	LD	L/B	3
9, F	–	–	–	L/B	1
10, F	–	–	–	L	2
11, M	–	–	LD	L	3
12, M	–	–	–	L	1
13, F	–	–	–	L	1

*Conventional MR: CC, thinning of corpus callosum; WMHs, white matter hyperintensities; VE, ventricular system enlargement; Clinical perinatal history: GE, gestational age at birth (weeks); CS, cesarean section; NIC, neonatal intensive care; Developmental milestones: MD, motor delay; LD, language delay. Comorbidity: L, language disabilities (expressive or mixed); M, motor disturbances; B, adaptive behavior disabilities; SES, socioeconomic status by Hollingshead Index of Social Position (1 lower, 2 lower–middle, 3 middle, 4 upper–middle, 5 Upper).*

### MRI PROTOCOL

Magnetic resonance imaging was performed using a 1.5 Tesla MR system (Siemens Magnetom Avanto, Erlangen, Germany). The MRI protocol consisted of: (1) a conventional MRI study including T2-weighted (TR/TE = 2920/22 ms; FoV = 240 mm, slice thickness = 4 mm, number of axial slices = 25) and fluid attenuated inversion recovery (FLAIR: TR/TE = 9000/121 ms; FoV = 240 mm, slice thickness = 5 mm, number of coronal slices = 24) scans to assess anatomical structure and to detect the presence of brain abnormalities; (2) High-resolution 3D T1 image (TR/TE = 1900/3.37 ms, matrix size = 192 × 256, in-plane resolution = 1×1 mm^2^, slice thickness = 1 mm, number of axial slices = 176) for VBM analysis.

Magnetic resonance anatomical and structural images obtained for all children were evaluated by an expert neuroradiologist blinded to the diagnosis.

Noteworthy, only 13 out of the 23 BIF children enrolled had MRI scan free from movement artifacts, while all 14 control children included in the study had good quality MRI scans.

All children were introduced to the MR protocol by a psychologist who trained them with play therapy and a mock scanner. During MRI all children had a video-goggle system to view movies.

### DATA ANALYSIS

We compared the BIF and TD groups on demographic, IQ level using the chi-square test and Mann–Whitney *U*-test for categorical and dimensional variables, respectively.

#### Voxel-based morphometry

Voxel-based morphometry with diffeomorphic anatomical registration using exponentiated lie algebra (DARTEL; Ashburner and Friston, 2000; Ashburner, 2007) was conducted for the image analysis as described in Taki et al. (2012) on 13 BIF and all TD children (10 BIF children were excluded due to motion artifacts). T1-weighted 3D MR images were analyzed using Statistical Parametric Mapping 8^1^ in Matlab (Math Works, Natick, MA, USA). GM, white matter (WM), and cerebrospinal fluid (CSF) were extracted and normalized using a Bayesian approach (Unified Segmentation; Ashburner and Friston, 2005), then these segmented tissue maps were used to create a custom, population-specific template using the DARTEL template creation tool (Ashburner, 2007), and all images were finally normalized to the MNI space. After spatial processing, statistical analyses were performed using SPM8 and VBM8 software^2^. Total intracranial volume (TIV) was included in the second-level analysis as nuisance variable. For exploratory purposes, non-hypothesized group differences were only considered if a cluster of 50 or more contiguous voxels exceeded an uncorrected *p* < 0.001 threshold at the local maximum voxel. We choose this permissive statistical threshold due to the small number of subjects included in this analysis (13 BIF and 14 TD children), and to the fact that no previous studies were available, limiting *a priori* hypotheses. The obtained clusters were then used to define the regions of interest (ROIs) for subsequent analyses.

#### Statistical analysis on ROI variables and IQ scores

We computed the number of GM voxels falling within each ROI for each subject, after thresholding the GM maps (75% of GM intensity value) by means of customized scripts. The obtained values represent numerical indices of GM volume for each subject, and were included in statistical analyses along with WISC-III indices (**Table 3**). Continuous variables were described using median and interquartile range according to variable skewness. Non-continuous variables were described by percentages. To reduce skewness and to rescale variables to a common scale the normit Blom (Blom, 1958) transformation was used. Finally, missing data (0.7% with sparse pattern between both control and analysis group) were imputed via the expectation maximization (EM) method (Dempster et al., 1977).

**Table 3:** Descriptive statistics of outcomes variables by group.

	Group TD *N* = 14		Group BIF *N* = 13	
Variable	Voxel *n* Median	Interquartile range	Voxel *n* median	Interquartile range
LPC-ROI1	211.5	[205.25–217.5]	221	[209–237]
LSM1-ROI2	76.5	[66.5–100.75]	148	[126–156]
RPTC-ROI3	85.5	[64.25–95.75]	127	[117–150]
RSM1-ROI4	17.5	[13–27.5]	40	[30–50]
RMOC-ROI5	130	[97–162]	127	[110–146]
LMOC-ROI6	58	[49–60]	50	[42–60]
RPH-ROI7	328	[325–343]	309	[296–330]
FSIQ	118	[107–120]	80	[77–83]
PIQ	116	[105–118]	82	[79–85]
VIQ	113.5	[103.25–118.25]	84	[77–86]
VCI	117	[102.75–119]	85	[76–87]
POI	117.5	[107–122.5]	85.5	[84.5–92.25]
FDI	101.5	[93.25–108.25]	85	[75–91]
PSI	103	[95.5–106]	74	[70.25–77.5]

*ROI, region of interest; voxel n median, number of voxels included in the ROI median value; LPC, left posterior cingulated; LSM1, left sensorimotor cortex; RPTC, right posterior temporal cortex; RSM1, right sensorimotor cortex; RMOC, right middle occipital cortex; LMOC, left middle occipital cortex; RPH, right parahippocampal gyrus; FSIQ, full scale IQ; PIQ, performance IQ; VIQ, verbal IQ; VCI, verbal comprehension index; POI, perceptual organization index; FDI, freedom from distractibility index; PSI, processing speed index.*

Both univariate and multivariate generalized linear model analysis of variance were used to investigate to what extent the group influenced GM volume. The multivariate test for the Hypothesis of No Overall Group Effect was reported using Wilks’ Lambda test. Least Square means and 95% confidence limits of normalized scores, *R*-square and *p*-values are reported in **Table 4**.

**Table 4:** Univariate and multivariate analysis of variance on magnetic resonance data.

MR variables (*)	TD children LS-mean [95% CL]	BIF children LS-mean [95% CL]	*R*-square	*p-value*
LPC-ROI1	–0.27 [–0.79; 0.024]	0.29 [–0.24; 0.83]	0.0893	0.1300
LSM1-ROI2	–0.56 [–0.99; –0.13]	0.61 [0.16; 1.05]	0.3765	*0.0007*
RPTC-ROI3	–0.69 [–1.05; –0.32]	0.74 [0.37; 1.12]	0.5638	*<0.0001*
RSM1-ROI4	–0.64 [–1.03; –0.25]	0.69 [0.28; 1.09]	0.4844	*<0.0001*
RMOC-ROI5	–0.01 [–0.55; 0.53]	0.01 [–0.55; 0.58]	0.0001	0.9550
LMOC-ROI6	0.17 [–0.37; 0.70]	–0.18 [–0.74; 0.38]	0.0330	0.3647
RPH-ROI7	0.50 [0.04; 0.95]	–0.54 [–1.01; –0.06]	0.2938	*0.0035*

MANOVA Results	Wilk’s λ	*F*-statistic	Degrees of freedom	*p-value*
	0.173	12.94	7	*<0.0001*

(*) Blom’s normit score of MR (magnetic resonance) variables; ROI, region of interest; LPC, left posterior cinguate; LSM1, left sensorimotor cortex; RPTC, right posterior temporal cortex; RSM1, right sensorimotor cortex; RMOC, right middle occipital cortex; LMOC, left middle occipital cortex; RPH, right parahippocampal gyrus.

**FIGURE 1:**
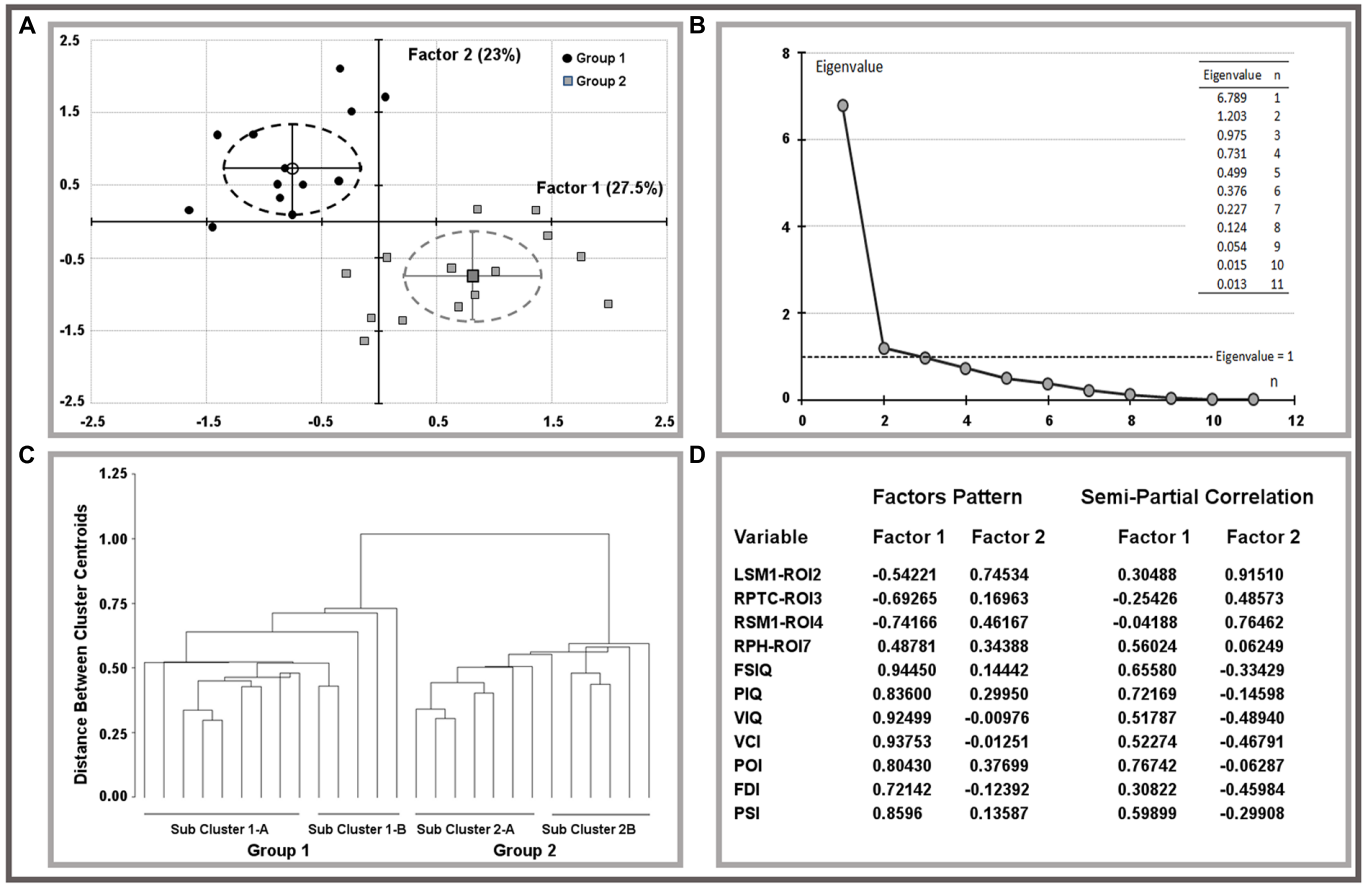
**Results of factor analysis on ROI variables and IQ scores.** Biplots of factor scores by group **(A)** and related multivariate analysis **(B,C)**. Explained variances and factor pattern were reported on **(D)**. LSM1, left sensorimotor cortex; RPTC, right posterior temporal cortex; RSM1, right sensorimotor cortex; RPH, right parahippocampus; FSIQ, full scale IQ; PIQ, performance IQ; VIQ, verbal IQ; VCI, verbal comprehension index; POI, perceptual organization index; FDI, freedom from distractibility index; PSI, processing speed index.

The oblique equamax factor analysis (Yaremko et al., 1986) of statistically significant ROI variables and IQ scores was used according to the hypothesis of a non-null correlation between factors to explore variable correlations and clusters among children. The Guttman–Kaiser criterion (Guttman, 1954) was used to determine the number of factors to retain after principal component analysis (PCA). Biplots of retained factors, variances explained, and variables loadings on rotated factors are reported in **Figure 1**, groups are described by means of factor’s centroids, 95% confidence limits of centroids are reported using ellipsoids of factor’s scores. Sub-cluster within groups were obtained using the un-weighted pair-group centroid method and reported using a tree-plot (**Figure 1**).

All statistical evaluations were performed using the SAS software package version 9.2., statistical tests were two-tailed and the alpha value of 0.05 was considered.

## RESULTS

### DEMOGRAPHIC CHARACTERISTICS, INTELLECTUAL FUNCTIONING, AND CLINICAL HISTORY

Children with BIF were not significantly different from TD children on the matching variable of age and sex (**Table 1**). Significant differences between the two groups were found in SES status by means of Hollingshead Index of Social Position (**Table 1**). History of perinatal incidences was positive in five BIF children (two children were born preterm, one of them had also cesarean section (CS) for oligohydramnios, while three had cesarean section due to preeclampsia with intensive care unit recovery in one case, for umbilical cord around neck with first Apgar 3/8 in one case and for abruptio placentae in the last), while eight had a history of delay and/or abnormal development of language and/or motor abilities (**Table 2**).

The assessment of learning abilities showed that all children with BIF presented with deficits in one or more areas: seven children in reading, three in both reading and writing, and three in the calculation area alone. Specific learning disability diagnosis was excluded because of the low general cognitive profile of the children (see **Table 1**). Finally, all BIF children showed a range of comorbidities involving motor skills, language abilities, and adaptive behaviors. Perinatal and developmental history was negative in all TD children, with regular pregnancy and partum, and normal developmental milestones.

### MR ANATOMICAL DATA RESULTS

Magnetic resonance anatomical and structural images obtained from 13 BIF and 14 TD revealed the presence of brain abnormalities in three BIF children. One child presented with a thinning of the splenium and the body of the corpus callosum, one had three non-specific small (2–3 mm) white matter hyperintensities (WMHs) in T2 and FLAIR images, and the last child presented with an enlargement of the ventricular system, not diagnosable as hydrocephalus. Given the nature of the brain alterations found in BIF children (minimal for dimension and involving selectively white matter) we decided to include them in the VBM analysis (that involved only GM, after segmentation of other brain tissues) after scrutiny of the accuracy of segmentation procedure. MR acquisition in TD children did not find any sign of brain abnormality.

### VBM RESULTS

**FIGURE 2:**
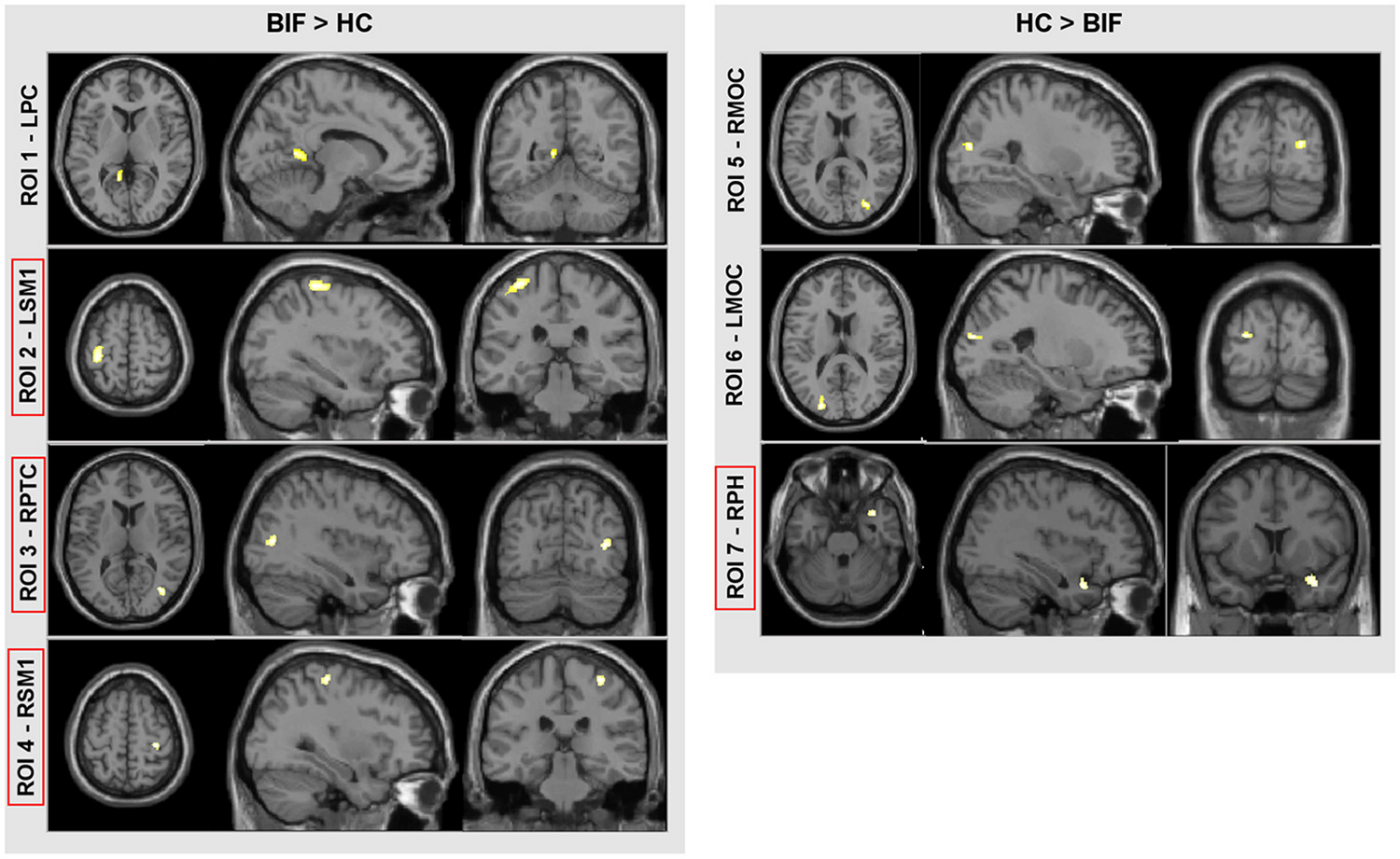
**VBM results.** Direct comparison between BIF and TD. In red squares, cortical areas confirmed by the univariate test for group effect (see “Materials and Methods” for further details and statistical thresholds). ROI, region of interest; LPC, left posterior cingulate; LSM1, left sensorimotor cortex; RPTC, right posterior temporal cortex; RSM1, right sensorimotor cortex; RMOC, right middle occipital cortex; LMOC, left middle occipital cortex; and RPH, right parahippocampus.

Voxel-based morphometry analysis showed differences in GM volumes in several areas (**Figure 2**; **Table 4**) in BIF compared to TD children; these areas were included in the subsequent ROIs analysis as described in the methods’ section. In particular, BIF versus TD showed: (1) Increased GM volume in left posterior cingulate (LPC-ROI 1, –11, –52, 6 x, y, z MNI space; *k* = 357; *z* = 4.24), left sensorimotor cortex (LSM1-ROI2, –33, –30, 63 MNI space; *k* = 1306; *z* = 3.89), right posterior temporal cortex (RPTC-ROI3, 38, –72, 10 x, y, z MNI space; *k* = 246; *z* = 3.74) and right sensorimotor cortex (RSM1-ROI4, 33, –28, 61 x, y, z MNI space; *k* = 327; *z* = 3.64; 2) decreased GM in right middle occipital cortex (RMOC-ROI5, 29, –81, 15 x, y, z MNI space; *k* = 239; *z* = 4.17), left middle occipital cortex (LMOC-ROI6, –24, –87, 16 x, y, z MNI space; *k* = 276; *z* = 3.27), right parahippocampal gyrus (RPH-ROI 7, 36, 11, –27 x, y, z MNI space; *k* = 700; *z* = 3.64).

### RESULTS OF STATISTICAL ANALYSIS ON ROI VARIABLES AND IQ SCORES

Both MRI (ROIs data) and IQ (IQ scores) data were rather skewed and highly inter-correlated, descriptions are reported in **Table 3**. The univariate test for group effect resulted statistically significant for LSM1-ROI2, RPTC-ROI3, RSM1-ROI4, and RPH-ROI7, but not for LPC-ROI1, RMOC-ROI5, and LMOC-ROI6 (**Table 4**). When considering all ROI variables in the MANOVA analysis, a statistically significant group effect was also found (*p* < 0.0001, Wilk’s λ = 0.173, *F* = 12.94, DF = 7). Finally, according to Guttman–Kaiser criterion, two principal components were retained when both statistically significant ROI and IQ variables were considered in PCA (**Figure 1**). After oblique equamax factor analysis, ROI, and IQ variables were found to be well associated with the first two factors resulting in a cumulative explained variance of 50.5%. These factors discriminated children according to group, no other sub-clusters were found when considering covariates. Within groups sub-clusters obtained by un-weighted pair–group centroid cluster analysis were not significantly associated with any of the first two rotated factors. Factor 1 was positively correlated with all IQ scores and was principally related to full scale IQ (FSIQ), verbal IQ (VIQ) and verbal comprehension index (VCI), having factor loadings of 0.94, 0.93, and 0.92, respectively. Moreover, Factor 1 was also found to be inversely related to LSM1-ROI2 (factor loading = –0.54), RPTC-ROI3 (factor loading = –0.69), and RSM1-ROI4 (factor loading = –0.74), and positively related to RPH-ROI7 (factor loading = 0.49), showing a close relationship between brain size in different regions and intelligence. According to these findings we can then deduct that the increment in GM volume in bilateral sensorimotor cortex (LSM1-ROI2; RSM1-ROI4) and in RPTC (ROI3) is associated with lower intelligence, while increased GM volume in RPH (ROI7) is associated with higher intelligence. Differently, Factor 2 was positively correlated with all ROI data and was principally related to LSM-ROI2 and RSM-ROI4, having factor loadings of 0.74 and 0.46, respectively. Moreover, Factor 2 was also found to be positively correlated with FSIQ (factor loading = 0.14), performance IQ (PIQ: factor loading = 0.30), and POI (factor loading = 0.38) and slightly negatively correlated with verbal subscales (VCI factor loading = –0.013 and VIQ factor loading = –0.010). This second factor illustrates how the differential components of the intelligence (verbal *versus* performance related functions) are influenced by brain maturation (**Figure 1D**).

## DISCUSSION

Data herein demonstrate the presence of abnormal GM development in BIF children with learning disabilities that correlates with IQ levels.

The results of VBM analysis offer important additional data unraveling that BIF with learning disabilities is associated with abnormal cortical and subcortical GM development. Our data showed that BIF children, compared to controls have increased regional GM volume in bilateral sensorimotor and right posterior temporal cortices and decreased GM volume in the right parahippocampal gyrus. It is frequent observation that children with intellectual disabilities (ID) also show motor development disabilities and/or delays with a high correlation between motor and executive functions (Hartman et al., 2010), and between the degree of ID and performance of manual dexterity (Vuijk et al., 2010).

The close relationship between motor skills and IQ development was first argued by Piaget (1984), whose studies linked the development of thought with the emergence of skilled action. More recently, a study (Wassenberg et al., 2005) on a large cohort of TD children found a positive correlation between motor and cognitive abilities such as WM, verbal fluency, visuomotor abilities. Moreover, motor and intellectual skills share some neural substrates in the prefrontal cortex and the neocerebellum, which are strictly correlated in terms of phylogenetic development (Diamond, 2000) and of activation during motor and cognitive tasks (Raichle et al., 1994). A recent longitudinal study on teenagers (Ramsden et al., 2011) showed that changes over time in verbal IQ (VIQ) correlated with changes in the left motor cortex. Finally, a positive correlation between visuomotor abilities and IQ score, and between intelligence and GM volume in TD children, has recently been demonstrated (Pangelinan et al., 2011). These data are in line with our results showing that BIF children have increased GM volume in the posterior temporal cortex, a region that is part of the visual dorsal stream whose role is to mediate the visual control of skilled actions such as grasping (Milner and Goodale, 2008).

All these evidences highlight the importance of motor skills and visuomotor integration in the development of intelligence. Our study provides the first neuroanatomical demonstration of this link by showing that motor and visuomotor cortices are “abnormally” developed in children with BIF.

An additional result of our analyses concerns the parahippocampal gyrus, an area that showed an increment in size in the TD group. The parahippocampal gyrus and other areas of the medial temporal lobe have generally been implicated in episodic memory formation and learning (Paller and Wagner, 2002; Phelps, 2004) and learning processes in which the social and emotional context is crucial (Bhatt et al., 2012). Our results, showing decreased volume in the parahippocampal gyrus in our sample of BIF children, can shed some light on the neural mechanisms of learning difficulties and lack of social competencies typical of BIF children. Interestingly, a correlation between hippocampal volume and SES has been recently demonstrated Noble et al. (2012). This relation is probably due to the association of lower SES with higher exposure to stress (Buss et al., 2007; McEwen and Gianaros, 2010; Tottenham and Sheridan, 2010). SES is indeed a well-known factor associated with the BIF condition (Karande et al., 2008; Hassiotis et al., 2011) and also in our cohort this factor was significantly different between the two groups.

A novel and relevant aspect of these data is the integration of brain volumetric and clinical data related to the IQ. Results of the PCA statistical analysis revealed that two factors were able to discriminate the groups (TD versus BIF). The first factor was inversely related to the size of left and right sensorimotor cortices and posterior temporal cortex, positively related to the size of the parahippocampal gyrus and all IQ indices. This factor can be interpreted as related to the cortical and subcortical brain development. During childhood and adolescence the cortical and subcortical components of the brain change dramatically (Rosenzweig, 2003; Wilke et al., 2003; Lenroot and Giedd, 2006; Groeschel et al., 2010; Silk and Wood, 2011) with regionally specific, age-dependent variations (Wilke and Holland, 2003; Sowell et al., 2004). In fact, brain development is a dynamic process characterized by regressive, synapse elimination (pruning), and progressive changes, such as arborization, synaptogenesis, and an increase in pyramidal cell somata, influenced by genetical, epigenetical (environmental and experience-related) factors that determine cortical thickening or thinning (Cowan et al., 1984; Huttenlocher and Dabholkar, 1997; Rosenzweig, 2003; Juraska and Markham, 2004; Petanjek et al., 2008; Petanjek and Kostović, 2012). Sowell et al. (2004) demonstrated that, during childhood, the dorsal frontal region shows cortical thinning while perisylvian and temporal lobe GM structures, such as amygdala and hippocampus, increase in volume. According to these evidences we interpreted our data as due to delayed thinning mechanisms in sensorimotor and posterior temporal cortex, and delayed cortical thickening mechanisms in parahippocampal cortex. Such findings are highly relevant for the comprehension of the motor and cognitive difficulties of children with BIF and reveal that the size of these cerebral regions was a good predictor itself of the IQ allowing for the discrimination between groups.

The second factor correlates positively with FSIQ and performance IQ scores and negatively with VIQ scores. In the field of theoretical model of intelligence, there is a common distinction, strongly supported by neurobiological data (Blair, 2006; Horn and McArdle, 2007), between the crystallized and fluid intelligence. Factor 2 shows how differential components of intelligence are influenced by brain maturation. It seems that IQ scores related to the commonly defined “fluid intelligence” (performance scores) are the mostly correlated with brain volumes and in particular with the sensorimotor cortex.

Our sample of BIF children has different risk factors: biological and environmental: three had a positive conventional MRI structural scan and five had a positive perinatal history. Notably, the two aspects coincide only in one child. In this context, the above mentioned MR results are important as they represent the first attempt, to our knowledge, to detect brain abnormalities as a possible cause of BIF and underlie the necessity to investigate this aspect even in the absence of positive perinatal histories.

In our sample we found both biological and environmental evidences that may have influenced maternal stress during pregnancy and emotional interactions during infancy. As recently demonstrated, epigenetic factors can affect brain development in the intrauterine life (Raznahan et al., 2012) but also until the third decade of life (Petanjek et al., 2011). Moreover it has been demonstrate that BIF is a condition highly associated with mental health problems and socioeconomic disadvantage (Hassiotis et al., 1999; Emerson et al., 2010). According to the characteristics of our sample and to the recent scientific evidences linking cortical development and epigenetics (Petanjek and Kostović, 2012), we think that our study can represent a first evidence that BIF with learning disability is associated with abnormal cortical development and this is relevant for the timing of rehabilitative intervention.

In conclusion, data herein represent the first attempt to relate MRI structural markers with the clinical spectrum of the BIF condition in a pediatric sample. The importance of our results lie in the fact that even in a small group of subjects with heterogeneous characteristics, it is possible to detect abnormal cortical development. This implies that, whatever the cause of BIF, brain development is affected. This is very important as it underlies the importance of early detection of this condition.

The small number of subjects and the clinical heterogeneity of the BIF sample constitute a limitation of our study, this should be considered a case study of a group of BIF. Our data, though, are quite clear and robust in showing a cortical development delay and a significant correlation between cortical development and IQ scores. For this reason we think our data demonstrate that, whatever the clinical condition, learning disabilities with BIF are associated with brain development abnormalities. Future longitudinal MRI studies will be useful in verifying whether these characteristics are modifiable over time and if rehabilitative interventions could drive the brain maturation toward patterns that resemble those seen in TD.

## Conflict of Interest Statement

The authors declare that the research was conducted in the absence of any commercial or financial relationships that could be construed as a potential conflict of interest.

## Footnotes

1 http://www.fil.ion.ucl.ac.uk/spm

2 http://dbm.neuro.uni-jena.de/vbm/
